# The Real Practice Prescribing Antibiotics in Outpatients: A Failed Control Case Assessed through the Simulated Patient Method

**DOI:** 10.3390/antibiotics12050915

**Published:** 2023-05-16

**Authors:** María Guadalupe Miranda-Novales, Karen Flores-Moreno, Mauricio Rodríguez-Álvarez, Yolanda López-Vidal, José Luis Soto-Hernández, Fortino Solórzano Santos, Samuel Ponce-de-León-Rosales

**Affiliations:** 1 Unidad de Investigación en Análisis y Síntesis de la Evidencia, Instituto Mexicano del Seguro Social, Centro Médico Nacional Siglo XXI, Mexico City 06720, Mexico; 2 Laboratorio de Microbioma, Facultad de Medicina, Universidad Nacional Autónoma de México, Mexico City 04510, Mexico; 3 Departamento de Microbiología y Parasitología, Facultad de Medicina, Universidad Nacional Autónoma de México, Mexico City 04510, Mexico; 4 Instituto Nacional de Neurología y Neurocirugía Manuel Velasco Suárez, Mexico City 14269, Mexico; joseluis_sotohernandez@yahoo.com; 5 Unidad de Investigación en Enfermedades Infecciosas, Hospital Infantil de México Federico Gómez, Secretaría de Salud, Mexico City 06720, Mexico; 6 Programa Universitario de Investigación Sobre Riesgos Epidemiológicos y Emergentes, Universidad Nacional Autónoma de México, Mexico City 04510, Mexico

**Keywords:** antibiotic management, primary care, health services research

## Abstract

The first level of medical care provides the largest number of consultations for the most frequent diseases at the community level, including acute pharyngitis (AP), acute diarrhoea (AD) and uncomplicated acute urinary tract infections (UAUTIs). The inappropriate use of antibiotics in these diseases represents a high risk for the generation of antimicrobial resistance (AMR) in bacteria causing community infections. To evaluate the patterns of medical prescription for these diseases in medical offices adjacent to pharmacies, we used an adult simulated patient (SP) method representing the three diseases, AP, AD and UAUTI. Each person played a role in one of the three diseases, with the signs and symptoms described in the national clinical practice guidelines (CPGs). Diagnostic accuracy and therapeutic management were assessed. Information from 280 consultations in the Mexico City area was obtained. For the 101 AP consultations, in 90 cases (89.1%), one or more antibiotics or antivirals were prescribed; for the 127 AD, in 104 cases (81.8%), one or more antiparasitic drugs or intestinal antiseptics were prescribed; for the scenarios involving UAUTIs in adult women, in 51 of 52 cases (98.1%) one antibiotic was prescribed. The antibiotic group with the highest prescription pattern for AP, AD and UAUTIs was aminopenicillins and benzylpenicillins [27/90 (30%)], co-trimoxazole [35/104 (27.6%)] and quinolones [38/51 (73.1%)], respectively. Our findings reveal the highly inappropriate use of antibiotics for AP and AD in a sector of the first level of health care, which could be a widespread phenomenon at the regional and national level and highlights the urgent need to update antibiotic prescriptions for UAUTIs according to local resistance patterns. Supervision of adherence to the CPGs is needed, as well as raising awareness about the rational use of antibiotics and the threat posed by AMR at the first level of care.

## 1. Introduction

Mexico’s health system is made up of public and private services. The former serves the majority of the country’s population, including formal workers and their families, as well as those who do not have any type of social security and cannot pay for private services, while the latter serves individuals through private insurance policies and those who can cover the costs of care with their own financial resources [1]. In recent decades, the use of private services has grown considerably. While in the year 2000, the number of visits to private medical doctors (MDs) represented 31% of the total number, this proportion increased to 37.6% and 38.9% in 2006 and 2012, respectively [2,3,4]

For health services, almost two decades ago, medical offices adjacent to chain pharmacies (MOAPs) began operating, mainly oriented to the sale of generic drugs for the low- and middle-income populations. These MOAPs can be classified according to the type of pharmacy to which they are linked: medical offices adjacent to independent pharmacies (MOAIPs) or to business chain pharmacies (MOABCPs) [5].

In 2013 it was estimated that nearly 13,000 MOABCPs treated 10 million patients per month and employed 32,500 physicians [6]. In 2016, the number of medical offices increased to 16,000, and according to the 2012 National Survey of Health and Nutrition (ENSANUT 2012), the visits to MOABCPs represented 41.5% of all private medical consultations in the country [4].

Until August 2010, the public sale of antibiotics in Mexico had no restrictions, and any person could buy them in pharmacies [7]; after that date, the obligation to obtain a medical prescription in order to purchase antibiotics came into force, contributing to an increase in the number of MOAPs. The main objective of the MOAP business model is to increase pharmacy sales, so the professionals who work in them are also called ‘point of sale doctors’ [8,9,10]. There is some concern regarding the quality of care at MOAPs, but most users report that it is good or even very good, with several advantages: low cost, convenience and short waiting times [11]. There is also uncertainty about the working conditions (obligations, rights and benefits) [9] which have been indicated as key elements directly related to patient care and safety [12].

The Federal Commission for Protection against Sanitary Risk (*Comisión Federal para la Protección contra Riesgos Sanitarios*, COFEPRIS) is the national regulatory authority in matters of health and is responsible for monitoring compliance with regulations regarding health risks for health service providers, as well as manufacturers and all those involved in the distribution and sale of medicines and medical devices, among other functions. From November 2013 to December 2017, COFEPRIS made 11,941 verification visits to MOAPs, resulting in 480 suspensions, equivalent to 4% of the verified establishments. At these verification visits, there was no mention of supervision of antimicrobial prescription according to clinical diagnoses [13].

The simulated patient (SP) method was developed by Barrows in 1968 for teaching purposes to assess the competence of medical doctors in a safe setting by means of a person trained to represent a disease [14,15]. It has been used in studies to assess the quality of care and decision-making in common clinical settings in several countries [16,17,18,19,20,21,22,23,24,25,26,27,28,29], and there are also useful guidelines for the reporting of research works in which this methodology is used [21]. 

The behaviour regarding the prescription of antimicrobials in MOAPs is not clear. The SP methodology can be used to evaluate prescriptions for the most common reasons for consultation leading to the prescription of antibiotics in ambulatory care, and it is also possible to measure adherence to clinical practice guidelines (CPGs).

The objective of this study was to record the prescriptions (type of antimicrobial, dose, route of administration and duration of treatment) in MOAPs in Mexico and to compare them with the CPGs for three clinical scenarios in adults: AP, AD and UAUTIs in adult women, represented by SP.

## 2. Results

A total of 280 clinical consultations with SP were given by different physicians. The distribution of medical consultations was: 101 (36.1%) for AP, 127 (45.4%) for AD and 52 (18.6%) for UAUTI. Most of the interactions took place at MOABCPs (238/280) and the rest in MOAIPs.

One hundred fifty-eight (56.4%) of the physicians were women and 122 (43.6%) were men. We found no differences in the pattern of antibiotic prescription between the sexes. The average seniority in the exercise of the profession of the consultant MD (calculated based on the year of issuance of their professional license) was 13.6 years. In terms of years of practice, 70 (25%) MDs had more than 20, 30 (10.7%) had between 10 and 19 and 166 (59.3%) had less than 10. The professional licenses of 14 MDs (5%) could not be found in the National Registry of Professions.

The median duration of the clinical interactions was 11 min (min: 7, max: 60). Diagnoses were given to all SPs along with their prescriptions, and all diagnoses were consistent with the condition simulated by the SPs. The median number of drugs in each prescription was 2 (minimum (min): 1; maximum (max): 5). Regarding the cost of the services rendered, 3 MOABCPs offered ‘free medical guidance’ at no cost; for the rest, the mean cost of the consultation was 2.0 USD (min: 1.5, max: 3).

A complete medical history and physical examination were performed in 174 (62.1%) of the clinical interactions, and in 106 of them (37.9%), the consultation focused on the current condition, and a minimal physical examination was performed. For the 101 AP scenarios, in 90 cases (89.1%), one or more antibiotics or antivirals were prescribed. The antibiotic group with the highest prescription pattern were aminopenicillins and benzylpenicillins (ampicillin, amoxicillin, amoxicillin/clavulanate, procaine and benzathine penicillin) with 30/90 prescriptions; followed by macrolides (erythromycin, azithromycin and clarithromycin) in 17/90 prescriptions and cephalosporins (cefalexin, cefuroxime and ceftriaxone) in 12/90 prescriptions. The group of antibiotics least prescribed for this clinical entity was the quinolones (norfloxacin, ciprofloxacin and levofloxacin). In 6/25 prescriptions for amantadine, an antibiotic was also prescribed (aminopenicillin) (Table 1). In 4/90 antibiotic prescriptions, a combination of an aminopenicillin plus a macrolide was indicated. In three SPs, although the diagnosis was bacterial pharyngitis, they did not receive antibiotics. For the rest of the SPs, the diagnoses were acute rhinopharyngitis (n = 35 (34.6%)), viral pharyngitis (n = 32 (31.6%)) or acute pharyngitis (n = 31 (30.6%)). In one SP, one MD used a delayed prescribing strategy (with a waiting period of 48 h), but the antibiotic to be used in case the symptoms persisted or worsened as ciprofloxacin.

For the 127 AD scenarios, in 104 (81.9%), an antibiotic and/or antiparasitic drug was prescribed (Table 2). Four (3.9%) SPs received one antibiotic plus an antiparasitic drug or one of those antibiotics referred to as ‘intestinal antiseptic’ (nifuroxazide and neomycin) simultaneously; 23 (22.1%) did not receive an antibiotic, but for 13 of them, the diagnosis was different to AD: colitis (n = 6), inflammatory bowel disease (n = 4) or peptic acid disease (n = 3). Antiparasitic drugs included: albendazole, mebendazole, metronidazole, quinfamide, nitazoxanide and diiodohydroquinone.

For the 52 SPs simulating UAUTIs, in all but one, an antibiotic was prescribed (Table 3); in the case of the SP who did not receive antibiotics, the doctor requested urinalysis with a microbiological culture before prescribing an antibiotic. Quinolones were the most prescribed antibiotic, followed by nitrofurantoin and TMP/SMZ. In six SPs, two antibiotics were indicated: nalidixic acid plus norfloxacin; nalidixic acid plus cephalexin; TMP/SMZ plus nitrofurantoin; nalidixic acid plus gentamicin; nalidixic acid plus amikacin and ciprofloxacin plus nitrofurantoin.

### Costs

**Figure 1:**
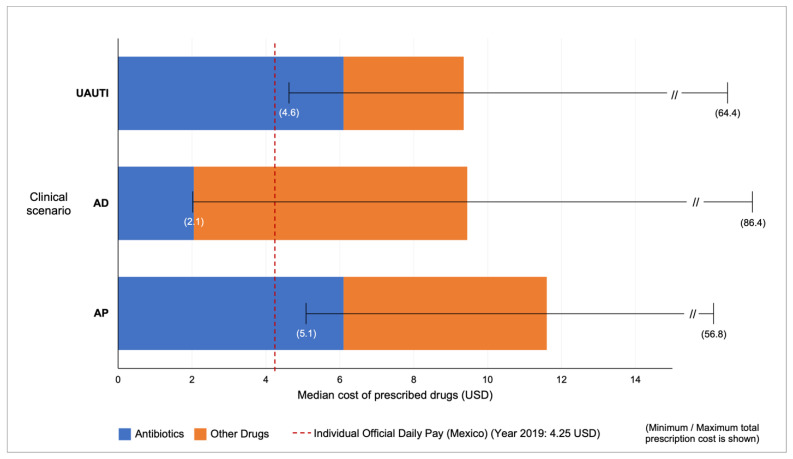
Cost of antimicrobials (antibiotics, antivirals, antiparasitic) and other drugs prescribed in the clinical scenarios: uncomplicated acute urinary tract infections (UAUTI), acute diarrhoea (AD) and acute pharyngitis (AP).

The median total costs per prescription were similar for the three scenarios. The highest cost was for AP (median: 11.6 USD), followed by AD (median: 9.5 USD) and UAUTI (median: 9.4 USD (Figure 1).

A great and diverse variety of other drugs not included or recommended in the CPGs were prescribed, such as mucolytics, expectorants, cough suppressants, steroids, bronchodilators, vitamins, centrally acting analgesics, anti-inflammatories, proton-pump inhibitors, lactobacilli, dopamine antagonists, antidiarrhoeal drugs (kaolin–pectin) and urine acidifiers. All these drugs contributed substantially to the final amount of each prescription, particularly for the AD scenario (Figure 1). Drugs prescribed in each interaction had a median of three (minimum 1-maximum 6). In the AP scenario, polypharmacy (5 or more drugs) was common and very diverse (16/101); examples of the combinations were as follows: loratadine/betamethasone plus nimesulide plus dextromethorphan plus vitamin C plus the antibiotic; ibuprofen/paracetamol plus amantadine plus dextromethorphan plus vitamin C plus the antibiotic; naproxen/paracetamol plus loratadine/betamethasone plus ambroxol plus the antibiotic.

## 3. Discussion

Despite clear recommendations in international and national guidelines for upper respiratory infections (URIs) (acute rhinopharyngitis (AR) or the common cold (CC)) and gastrointestinal diseases (acute diarrhoea (AD)), antibiotics are frequently prescribed for these conditions in outpatient settings [22,23,24,25]. Fleming-Dutra et al. [26] estimated the annual appropriate antibiotic prescription rate in the US for URIs. In all, acute respiratory conditions per 1000 population led to 221 antibiotic prescriptions (95% CI, 198–245) annually, but only 111 antibiotic prescriptions were estimated to be appropriate. In India, Kotwani et al. determined that during 2007 and 2008, patients with acute diarrhoea were prescribed at least one antibiotic in both public (171 of 398 (43%)) and private facilities (76 of 110 (69%)) [27], whereas the rate of antibiotic overuse in Thailand in adults with acute diarrhoea was 48.9% (86 of 176 patients) [28]. Appropriateness of antibiotic prescription is difficult to assess with certainty because important clinical data and risk factors are frequently lacking in the medical charts. The SP method makes it possible to gather relevant and real information on the quality of care. Satyanarayana S et al. summarised the experience of 3086 SP interactions in four countries representing cases of pulmonary tuberculosis, where providers prescribed medications that were unnecessary or harmful in 83% of the interactions; medications of interest were broad-spectrum antibiotics, fluoroquinolones and steroids [19]. In this study, volunteers were successful in representing the clinical scenario. Most of them received a diagnosis consistent with the signs and symptoms, so the training was adequate, and they appeared to be genuine patients. This strategy makes it possible to reproduce a situation in the daily life of an individual who goes to consultation and objectively obtains a result identical to that obtained with a real patient.

In Mexico, in May 2010, it was established by law that the sale and dispensing of antibiotics in private pharmacies may only be carried out with the presentation of a prescription [29]. Before this law was enacted, it was calculated that 40 to 60% of pharmacy customers self-medicated or asked for a recommendation from the pharmacy employee. After the law came into effect, the pharmacies, especially chain pharmacies, set up adjacent offices to offer very low-cost and free consultations, thus providing customers with full service. These offices satisfy a demand from the population, and the service they offer is considered satisfactory by users due to promptness and accessibility, while the pharmacy can continue to sell antibiotics. A study conducted in 1996 evaluated the prescriptive behaviour of pharmacy attendants, with simulated clients representing a gonorrhoea scenario. The attendants with no medical training prescribed adequate treatment in only 25% of the cases [30]. Researchers analysed the consumption of medicines in clients of private pharmacies and found that both by self-medication and by prescription, a wide variety of medicines were purchased: analgesics, antitussives, antibiotics, vitamins, herbal remedies, antidiarrhoeals, antiparasitic drugs and antihistamines, several of which in combinations that are not recommended [31]

Unfortunately, the use of antibiotics for the two most common causes of consultation (acute respiratory infection and acute diarrhoea) is like that reported in developing countries. In a systematic review by Li et al., the overall average of antibiotic prescriptions for upper respiratory tract infections was 83.7% (95%CI 80.6–86.4%) [32]. In our study, in the acute rhinopharyngitis scenario, all doctors made a correct diagnosis, but an antibiotic was prescribed in 9 out of 10 interactions. Most of the doctors who provided the consultations had less than 10 years of practice, so it is possible that this group was more familiar with the use of CPGs. Due to the design of the study, it was not feasible to explore this issue. In recent years, the management of CPGs has been a key point in the training programs and is included in every curriculum; current generations may be more familiar with its existence and use. However, some factors have been associated with antibiotic prescription, such as diagnosis of acute bronchitis, symptoms observed in the physical exam (fever, purulent sputum, tonsillar exudate) and the physician’s perception of the patient’s desire for antibiotics [33] In this study, all volunteers were healthy individuals and did not demand a prescription for an antibiotic. In addition to antibiotics, other prescribed drugs did not have clinical indications, such as antivirals, expectorants, mucolytics, bronchodilators and steroids.

In the acute diarrhoea scenario, our finding of an 82% antibiotic or antiparasitic prescription rate was higher than in other countries such as India [27]; a study conducted in New Delhi reported a prescription rate for antibiotics of 43% in the public sector and 69% in the private sector, in which the most commonly prescribed antibiotics were quinolones, followed by cephalosporins (cefuroxime and cefalexin) and in third place doxycycline. Increased prescribing in the private sector and lack of adherence to clinical practice guidelines are emphasised. In our study, the most prescribed drugs were TMP/SMZ and antiparasitic drugs, which are relatively inexpensive, but the final cost increases with all the medications added to the prescriptions.

Finally, the most common antibiotics prescribed for UAUTI in women were quinolones. The Mexican CPGs [24] note that these drugs should not be used as first-line treatment; however, it is stated that if there is no response to the drug initially indicated (nitrofurantoin or TMP/MSZ), the alternative is ciprofloxacin. Since it is accepted that it is unnecessary to take a urine culture before the antibiotic prescription, the aetiology would not be available to indicate a specific treatment in case of failure. Our volunteers have not prescribed the short-course treatment recommended in the guideline, which can significantly decrease the number of days of antimicrobial treatment.

If antibiotics are prescribed unnecessarily or used inappropriately, they can cause toxicity and other adverse effects, such as allergic reactions, gastrointestinal problems, headaches, and neurologic symptoms [34]. The patients or the institution that covers their care incurs additional expenses. Moreover, the inappropriate use of antibiotics by unlicensed individuals can contribute to the development of antimicrobial resistance. We found a non-negligible number (n = 14 (5%)) of physicians in the MOAPs practicing with a license that could not be verified as valid in the official databases. In these cases, the national regulatory authority (COFEPRIS) should suspend the activities of these medical offices, and those responsible must be held accountable.

Our study has several limitations. The number of interactions may seem small, but according to the sample size estimate, increasing the number of consultations probably would not lead to a different result. The assumptions used considered an even lower percentage of antimicrobial prescriptions. Factors that may have influenced the prescription of antibiotics by the physicians are not considered, among others, the load of the number of consultations and the hour of the day. It seems to be that an important factor is the economic interest of service providers that coercively influence their medical employees to increase the number of drugs listed in a prescription. The other is the limited consultation time available, which can lead the doctor to prescribe an antibiotic instead of educating the patient and explaining why it is not required.

In conclusion, the SP method allows prescription practices to be evaluated with objectivity and the identification of areas of opportunity to improve adherence to the CPGs and update their content. There is low adherence to CPGs in the primary care settings evaluated. Antimicrobial overuse is a considerable problem in common acute conditions (AP and AD), and apparently, the most frequent self-limited viral aetiology, which does not require antibiotics, is not considered. In UAUTIs, current antimicrobial resistance in uropathogens is also not considered, and several microbiological failures can be expected with the extensive use of quinolones.

## 4. Materials and Methods

This was a descriptive cross-sectional survey in which healthy medical and chemistry students were invited to participate. After review and approval by the institutional review board, volunteers who gave their consent were trained to represent one of the three clinical scenarios: AP, AD or UAUTI, according to the clinical description of the corresponding national CPG (Table 4) [22,23,24].

### 4.1. Procedure

Twenty-four research assistants were trained to act as SPs according to a script for each clinical scenario (AP, AD, UAUTI) in which signs and symptoms were described according to clinical practice guidelines [22,23,24], All participants were in good general health and had no prior experience as SPs or with the method; 18 were men (median age 29 years; interquartile range (IQR): 23–29) and 6 women (median age 23 years; IQR: 21–27). UAUTI was addressed only by the 6 women. In this method, the SP is a person acting a role. The training to represent the clinical cases was carried out in three sessions of 30 min each, supervised by a physician with clinical experience who was part of the research team. After the sessions, each participant simulated consultation with the instructor physician to ensure that the representation was consistent with the clinical scenarios. The advantage of the method is that a simulation can be reproduced for multiple participants, and the same simulation can be replicated with different actors [14].

Between May 2018 and January 2019, the SPs were randomly assigned to different MOABCPs and MOAIPs. After each visit, the participants recorded information related to the care received: duration of the medical consultation, characteristics of the medical interview and physical examination, indications and prescriptions. Drug prices were obtained through a specialised online search engine that includes the prices of medicines for at least 12 different pharmacies. For each drug, the average cost was obtained, as well as the minimum and maximum. Costs are shown in US dollars (USD); all costs were obtained in Mexican pesos (MXN) and the average exchange rate of 2019 was used (1 USD = 20 MXN).

### 4.2. Sample Size

A calculation was made with the estimated number of MOAPs in Mexico City’s Metropolitan Area (n = 3360) [6] and the expected frequency of adequate antibiotic prescriptions for each scenario [23] as follows:

AP: 13% (95% CI, 7–22; confidence limit: 7%), a total of 86 visits.

AD: 9% (95% CI, 4–16; confidence limit: 5%), a total of 121 visits.

UAUTI: 75% (95% CI, 61–86; confidence limit: 10%), a total of 36 visits.

All the information from the registration forms of the medical consultations and prescriptions was captured in a database by the research group; the analysis of the information was performed through descriptive statistics with simple frequencies, percentages, median, interquartile range (IQR) and minimum and maximum values.

## Figures and Tables

**Table 1:** Antimicrobial prescriptions in 101 acute pharyngitis (AP) interactions.

Antimicrobial	n	%
Aminopenicillin/Benzylpenicillin	30	29.7
Macrolide	17	16.8
Cephalosporins	12	11.8
Quinolones	9	8.9
Clindamycin	1	0.9
Fosfomycin	1	0.9
Total with at least one antibiotic	70	69.3
Amantadine	25	24.7
No antibiotic	11	10.8

**Table 2:** Antimicrobial prescription in 127 acute diarrhoea (AD) interactions.

Antimicrobial	n	%
TMP/SMZ	35	27.6
Quinolones	26	20.5
Aminopenicillin	4	3.1
Cephalosporins	3	2.4
Aminoglycoside	3	2.4
Tetracycline	2	1.6
Chloramphenicol	1	0.8
Macrolide	1	0.8
Total with at least one antibiotic	75	59
At least one antiparasitic drug	28	22
‘Intestinal antiseptics’	14	11
No antibiotic	23	18.1

TMP/SMZ = trimethoprim–sulfamethoxazole.

**Table 3:** Antimicrobial prescription in 52 uncomplicated acute urinary tract infections (UAUTI) interactions.

Antimicrobial	n	%
Quinolones	38	73
Nitrofurantoin	8	15.4
TMP/SMZ	5	9.6
Cephalosporins	2	3.8
Aminopenicillin	1	1.9
Aminoglycoside	2	3.8
Macrolide	1	1.9
Total with at least one antibiotic	51	98
No antibiotic	1	1.9

**Table 4:** Standardised patient clinical case scenarios and accepted recommendations according to national CPGs (References [22,23,24]).

Clinical Scenario	Symptoms	Management
Acute pharyngitis (AP)	-Sore throat -Dry cough -Runny nose -Conjunctival irritation (no secretion) -No fever Onset of symptoms: two days	Symptomatic treatment: paracetamol 500 mg orally every 8 h for 3–5 days or ;non-steroidal anti-inflammatory orally every 12 h for 3–5 days
Acute diarrhoea (AD)	-Abdominal pain -Loose stools (4–5 per day without mucus or blood) -Occasional nausea -No fever Onset of symptoms: 1 day	Oral rehydration solutions Astringent diet Watch for alarm signs of dehydration
Uncomplicated acute urinary tract infection in adult women (UAUTI)	-Dysuria -Urinary frequency and urgency -Abdominal discomfort -No fever -Onset of symptoms: 2 days	1st line treatment: (a) trimethoprim/sulfamethoxazole 160 mg/800 mg, twice daily for three days or nitrofurantoin 100 mg twice daily for 7 days. If after 3 day symptoms persist, request urine culture and initiate ciprofloxacin 250 mg twice daily for 3 days.

## Data Availability

The data presented in this study are available on request from the corresponding author.
